# Safety signals reinforce instrumental avoidance in humans

**DOI:** 10.1101/lm.053914.123

**Published:** 2024-08

**Authors:** Courteney T.L. Fisher, Gonzalo P. Urcelay

**Affiliations:** School of Psychology, University of Nottingham, Nottingham NG7 2RD, United Kingdom

## Abstract

Safety signals reinforce instrumental avoidance behavior in nonhuman animals. However, there are no conclusive demonstrations of this phenomenon in humans. Using human participants in an avoidance task, Experiments 1–3 and 5 were conducted online to assess the reinforcing properties of safety signals, and Experiment 4 was conducted in the laboratory. Participants were trained with CSs+ and CSs–, and they could avoid an aversive outcome during presentations of the CSs+ by pressing their space bar at a specific time. If successful, the aversive outcome was not presented but instead a safety signal was. Participants were then tested—whilst on extinction—with two new ambiguous test CSs. If participants made an avoidance response, one of the test CSs produced the trained safety signal and the other was a control. In Experiments 1 and 4, the control was followed by no signal. In Experiment 2, the control was followed by a signal that differed in one dimension (color) with the trained safety signal, and in Experiment 3, the control differed in two dimensions (shape and color) from the trained safety signal. Experiment 5 tested the reinforcing properties of the safety signal using a choice procedure and a new response during test. We observed that participants made more avoidance responses to the ambiguous test CSs when followed by the trained signal in Experiments 1, 3, 4, and 5 (but not in Experiment 2). Overall, these results suggest that trained safety signals can reinforce avoidance behavior in humans.

Avoidance behavior is a hallmark of all anxiety disorders. Avoidance can be defined as behavior that prevents the onset of an aversive outcome (Dinsmoor 1977). In the context of social anxiety, for example, this could mean not going to social events and/or seeing certain people to prevent disapproval by others. Research within the anxiety field has largely focused on fear acquisition and extinction (Graham and Milad 2011; Urcelay 2012). This has been a success in terms of developing new treatments for anxiety disorders (e.g., exposure therapies); however, there is still a large relapse rate (Scholten et al. 2013) so researchers have been exploring new avenues of research, for example, targeting avoidance behavior (Urcelay and Prével 2019). One of the most well-known theories of avoidance is Mowrer's two-factor theory (Mowrer 1951), which argues that avoidance involves two components, the first being classical fear conditioning. It is these classically conditioned signals that elicit fear, which results in instrumental avoidance behavior reinforced by terminating the fear experience elicited by the signals. In other words, in this conceptualization, (classically conditioned) fear drives instrumental (avoidance) behavior. However, we know from subsequent research in rats (Mineka 1979) and humans (Vervliet and Indekeu 2015) that when fear has been extinguished, avoidance behavior persists. This suggests that fear alone does not always account for the persistence of avoidance behavior. A burgeoning question within this literature is concerned with what drives avoidance behavior—given that fear alone does not fully account for it. In recent years, this has led to a resurgence in interest on avoidance behavior to better understand what drives avoidance behavior and why it persists—particularly in human participants.

One of those theorized drivers of avoidance behavior is relief provided by safety signals. Relief is a pleasant emotion that is triggered by the omission of an expected aversive event (Vervliet et al. 2017). Therefore, when people avoid, they prevent an aversive stimulus which in turn results in relief (Denny 1971). Relief can be particularly difficult to study because it depends on participants actively avoiding. Researchers have therefore tried to infer the reinforcing nature of relief by testing whether stimuli paired with relief produced by the absence of an aversive event (safety signals) can reinforce instrumental avoidance (Fernando et al. 2014a).

Historically, the literature on avoidance and safety signals has predominantly focused on nonhuman animals and while this provides insights into avoidance behavior, it is unknown whether this translates to humans. In experiments by Fernando and colleagues (Fernando et al. 2014a) rats were trained in a free-operant avoidance task where two different lever presses (trained alone, on separate sessions) could result in avoidance of a foot shock, and responses were followed by a 5 sec auditory (safety) signal. Rats were then tested in sessions in which both levers were presented, but responses on only one of the levers were followed by the safety signal. They observed that safety signals increased avoidance behavior to the lever that was followed by the safety signal, both when the shock was presented during the session, and in a choice test conducted on extinction (i.e., in the absence of shocks). This suggests that the safety signals reinforced avoidance behavior, which is in line with prior evidence by Weisman and Litner (1969, see also Rescorla 1969 for evidence in dogs). They trained rats using a free-operant avoidance procedure in which rats had to turn a wheel to avoid shocks. Following this, rats experienced explicitly unpaired training in which a tone was uncorrelated with a shock, thus endowing the tone with inhibitory properties. After explicitly unpaired training of the tone, rats were re-baselined on the free-operant avoidance task, and behavior was reinforced according to a differential reinforcement of high (DRH) rates schedule, in which the inhibitor was presented when rats avoided at higher rates than in the baseline. Rats that received explicitly unpaired training avoided more than control rats trained with a CS/US random schedule. Following another re-baseline period, avoidance was now reinforced according to a differential reinforcement of low (DRL) rates schedule, in which the signals are presented when subjects responded at a lower rate than in the baseline. Again, rats slowed their responding more when avoidance was followed by the inhibitor relative to the control stimulus. The results revealed that safety signals trained in a Pavlovian explicitly unpaired procedure (CS−/US) were able to bi-directionally control avoidance behavior in rats (reinforcing either high or low rates of avoidance), further showing the reinforcing effects of the safety signal. Finally, Dinsmoor and Sears (1973) trained pigeons to avoid a foot shock by pressing a pedal, and presented a 1000 Hz tone immediately after a successful avoidance as a safety signal. During the test, they varied the frequency of the tone safety signal and observed generalization decrement of the safety signal's reinforcing properties. That is, lower avoidance responses were observed at the test when the safety signal was different in frequency (500 or 2000 Hz) from the trained safety signal (1000 Hz).

Although there is convincing evidence that safety signals can reinforce instrumental behavior in nonhuman animals, there is a dearth of convincing evidence in humans. In a report by Angelakis and Austin (2015), participants played a computer game in which they could gain or lose treasures by clicking on a map. While playing, participants could also press a pedal to avoid bombs (and point losses), and this avoidance resulted in the presentation of a blue bar on the screen. During test sessions, the bar was yellow and would turn to blue when participants pressed the avoidance pedal. Participants pressed the avoidance pedal more when the bar changed from yellow to blue than the opposite, presumably showing the reinforcing properties of the (blue) safety signal. However, the report only recruited six participants, did not counterbalance the identity of the safety signal (blue or yellow), and importantly did not provide any statistical tests in support for the descriptive findings, making it difficult to conclude that the safety signal was indeed reinforcing avoidance behavior.

Moreover, there have been studies in which humans could avoid a shock that was signaled by a predictive CS+, and were asked immediately after an avoidance response to subjectively rate the extent to which they felt “relief.” In line with the notion that avoidance responding provides relief, participants gave higher subjective ratings of relief following shock avoidance, in particular early in training (Vervliet et al. 2017). A second report replicated these findings and further revealed that subjective relief ratings were higher following avoidance responses to a CS+ than to the presentation of a CS− (San Martin et al. 2020). Finally, it has been observed that subjective relief was higher in participants with PTSD and Panic disorder relative to healthy controls (De Kleine et al. 2023). However, these studies in humans used self-reported measures of relief such as asking participants how much relief they experienced following a successful avoidance response. Therefore, despite the subjective measures suggesting that avoidance response results in relief, there is hitherto no behavioral evidence in humans that safety signals reinforce avoidance behavior in humans.

The aim of this study was to investigate whether safety signals can act as reinforcers in a human instrumental avoidance task. Unlike previous reports, during avoidance training, we presented a discrete safety signal following a successful avoidance response, and later assessed whether the safety signal reinforced avoidance responses to novel stimuli. Because presenting the aversive outcome during the test can result in new learning during the test, we developed a procedure in which participants were tested in extinction (no aversive outcome presented during the test session) and using novel stimuli. Of course, the problem with using novel stimuli during tests is that participants are unlikely to respond to the new stimuli. To overcome this, during training participants experienced a discrimination between two stimuli in which the angle dimension was the relevant one to solve the discrimination (CS+ 90°, CS− 0°, counterbalanced). During the test, we presented 45° and 135° stimuli, which are in between those used during discrimination training. Pilot experiments revealed when we used one CS+ (e.g., 90°) and one CS− (e.g., 0°) during training, the novel test stimuli (45° and 135°) were too novel and participants made little to no responses during the test. To facilitate the transfer, during training we used two sets of stimuli (CS+: 80°, 90°, and 100°; CS−: 350°, 0°, and 10°; counterbalanced) as we observed that this variability during training facilitated the observation of responses during the test session.

In other words, in this series of experiments, participants were trained to avoid the appearance of an unpleasant image (or loud noise) which was signaled by one stimulus (CS+), while a second stimulus (CS−) was not paired with the aversive outcome. CS+ and CS− were Gabor patches with variable (±10°) orientation lines but around either 0° or 90° (counterbalanced for CS+ and CS−; see Fig. 6). The unpleasant image (or loud noise) could be avoided by pressing the space bar on the keyboard. Critically, and following previous reports (Flores et al. 2018; Urcelay et al. 2024), in order to successfully avoid participants had to respond 1 sec before the US was scheduled to appear, and the exact time of appearance within the CS was variable, which leads to numerous responses per trial. When participants avoided the US, they were then shown the safety signal (an image on the screen) for 3 sec. During the test phase, the US was no longer presented; however, participants were not informed of this, and they were presented with new Gabor patches (45° and 135°). Due to the ambiguity of these new Gabor patches (the orientation was exactly in between the orientation values of the CS+ and CS−), participants could respond or not. One of the new Gabor patches (45°) was followed by the trained safety signal if participants responded, and the other (135°; counterbalanced) served as the control (this differed per experiment). In Experiment 1, during the test we measured avoidance responses to a Gabor patch that was followed by the trained safety signal and compared it with responses to one that was followed by nothing. In Experiment 2, we measured avoidance responses to a Gabor patch that was followed by the trained safety signal and compared it to a Gabor patch that was followed (if participants responded) by a somewhat similar safety signal (same shape but a different color from the blue–green color continuum). In Experiment 3, we measured avoidance responses to a Gabor patch that was followed by the trained safety signal and compared it to responses made to a Gabor followed by a dissimilar signal (differed both in shape and in color). In Experiment 4, we replicated Experiment 1 but conducted the experiment in the laboratory with a biologically relevant aversive outcome (i.e., a 95 dB tone). Finally, in Experiment 5, we used the same stimuli as Experiment 3 but, we changed the response in the test phase to a mouse click, so participants now saw both Gabor patches on the screen at the same time and had the choice to respond to either, by directing the mouse pointer and clicking on them.

## Results

### Experiment 1: Participants avoid more in the presence of stimuli that produce the safety signal compared to stimuli that produce nothing

The first experiment assessed whether response-produced safety signals reinforce avoidance behavior in humans. We first trained participants with two sets of Gabor stimuli (counterbalanced). One set (e.g., orientations 80°, 90°, and 100°) was paired with the aversive outcome whereas the other (e.g., orientations 350°, 0°, and 10°) was not. Participants could avoid the aversive outcome by pressing the space bar. If participants successfully avoided the aversive outcome, they experienced the safety signal (an aqua square). They were then tested in extinction with two new Gabor stimuli (orientations 45° and 135°), one of which produced a safety signal if participants avoided and the other did not (counterbalanced). If safety signals reinforce avoidance behavior, we expected participants to respond more to the Gabor patch that produced the safety signal despite both Gabors being novel and never paired with the aversive outcome. All *P*-values reported were adjusted for sphericity where needed.

**Figure 1.**
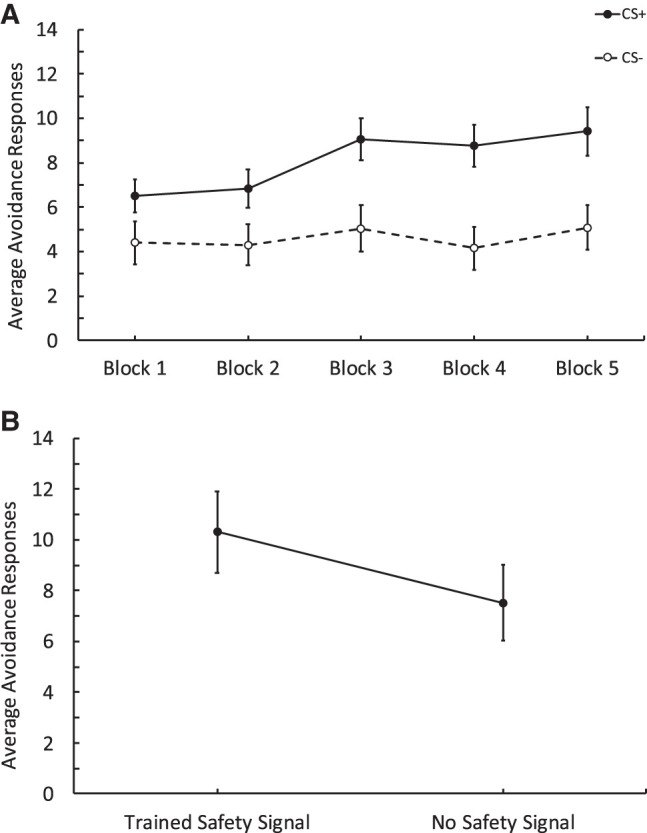
Results from Experiment 1 comparing avoidance responses to obtain a response-produced safety signal relative to no signal. (*A*) Training data depicting avoidance responses to the CSs+ and CSs−. Each block contains three trials. Participants made more responses to the CSs+ across blocks compared to the CSs−. (*B*) Test data showing avoidance responses to the Gabor that was followed by the trained safety signal versus responses to a Gabor that was followed by no signal. Participants made more responses to the trained safety signal than no signal. Error bars depict standard error of the mean.

Figure 1A shows that during training, participants (*n* = 72) responded more to the CSs+ than the CSs−. To assess this, we measured the rate of responding for each trial during the training phase. A repeated-measures ANOVA with CS (CSs+ vs. CSs−) and Trials (Trials 1–15) revealed a main effect of CS, *F*_(1,71)_ = 18.86, *P* < 0.001, $\eta_{p}^{2}$ = 0.210, trial, *F*_(6.78,481.54)_ = 4.78, *P* < 0.001, $\eta_{p}^{2}$ = 0.063, and a significant interaction, *F*_(8.41,597.1)_ = 3.85, *P* < 0.001, $\eta_{p}^{2}$ = 0.51, suggesting that as trials progressed, participants responded more to the CSs+ compared to the CSs−. We also analyzed the number of safety signals that participants experienced during the training phase by block (three trials per block; see Table 1 for descriptives). As training trials progressed, the number of safety signals received increased. A one-way ANOVA assessing the effect of Blocks (1–5) revealed a significant effect, *F*_(3.66,260.11)_ = 10.01, *P* < 0.001, $\eta_{p}^{2}$ = 0.124.

**Table 1.** Summary table of means and standard error of the number of safety signal presentations experienced during the training phase (blocks of three trials)

	Block 1	Block 2	Block 3	Block 4	Block 5
Experiment 1	1.51 (0.13)	1.40 (0.14)	1.96 (0.14)	1.99 (0.13)	1.9 (0.15)
Experiment 2	1.6 (0.11)	1.45 (0.13)	1.91 (0.15)	2.04 (0.15)	2.05 (0.16)
Experiment 3	1.46 (0.11)	1.36 (0.14)	1.92 (0.14)	1.99 (0.14)	2.09 (0.13)
Experiment 4	1.68 (0.18)	2.46 (0.13)	2.61 (0.09)	—	—
Experiment 5	1.24 (0.14)	1.39 (0.15)	1.46 (0.17)	1.63 (0.17)	1.63 (1.7)

Note that Experiment 4 contained nine training trials; therefore, there are only three training blocks instead of five.

Critically, during the extinction test, participants made more avoidance responses to the Gabor stimulus that was followed by the safety signal compared to the one that was not (see Fig. 1B). A repeated-measures ANOVA with Signal (Signal vs. No Signal) and Trials (1–8) compared avoidance responses to the Gabor followed by the safety signal versus the one which was not followed by a signal. There was a main effect of signal, *F*_(1,71)_ = 16.28, *P* < 0.001, $\eta_{p}^{2}$ = 0.187, revealing more avoidance responses to the Gabor followed by the safety signal, a marginal effect of trial in that responding decreased as trials progressed, *F*_(2.81,199.82)_ = 2.19, *P* = 0.095, $\eta_{p}^{2}$ = 0.030, but there was no interaction between these factors, *F*_(3.74,265.89)_ = 1.26, *P* = 0.285, $\eta_{p}^{2}$ = 0.018. Following the behavioral test, we assessed the expectancy data for the CS+ and the CS− and found that participants rated the CS+ more likely to be followed by the aversive stimulus than the CS−. A paired *t*-test revealed there was a significant difference in the expectancy scores for the CS+ (*M* = 6.38, SD = 2.146) and the CS− (*M* = 3.46, SD = 2.181); *t*_(71)_ = 8.8, *P* < 0.001, Cohen's *d* = 1.03.

Experiment 1 revealed that participants emitted more avoidance responses to an ambiguous Gabor patch which resulted in the safety signal relative to one which did not result in the safety signal. These findings suggest that the safety signal reinforced avoidance behavior. However, it could be possible that the results from Experiment 1 are due to any perceptual signal providing reinforcement, a phenomenon that has been named sensory reinforcement (Lovaas et al. 1987). Experiments 2 and 3 explored this possibility by using a control condition in which avoidance responses to the Control Gabor stimulus were followed by a stimulus that was to some extent perceptually similar to the trained safety signal (Experiment 2) or dissimilar to the trained safety signal (Experiment 3).

### Experiment 2: Participants avoid similarly during the test to a Gabor followed by a trained safety signal relative to one that is followed by new but similar signal

If the trained safety signal is reinforcing, then participants should respond to a Gabor when it is followed by a safety signal that has been trained compared to a Gabor that is followed by a new stimulus. In Experiment 2, participants received avoidance training with the same stimuli and procedure as that used in Experiment 1. The test was also the same, except that now one Gabor (45° or 135°, counterbalanced) was followed by the trained safety signal, and the control Gabor was followed by another signal that was of a different color (green or blue, counterbalanced), but similar in shape to the trained signal (i.e., square; see Materials and Methods and Fig. 7B for more details).

**Figure 2.**
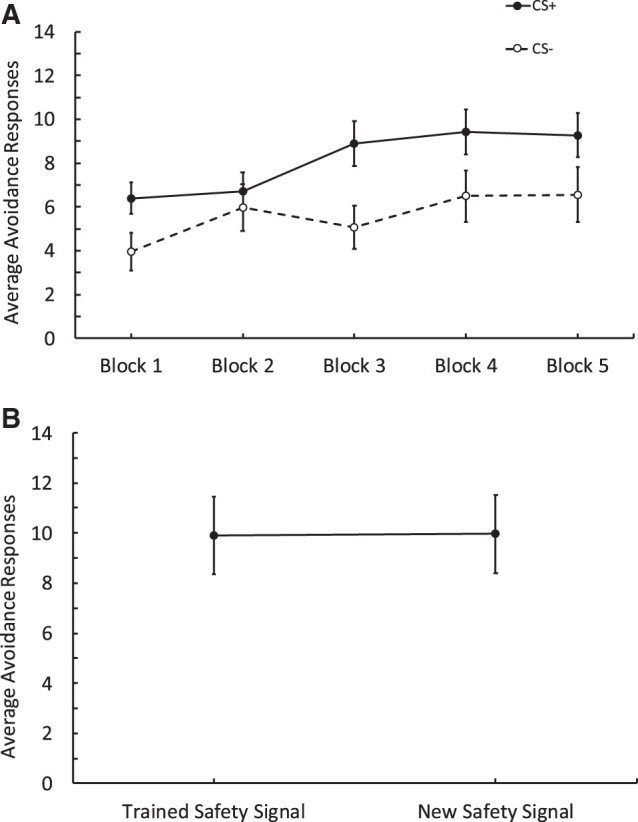
Results from Experiment 2 comparing avoidance responses to obtain a response-produced safety signal relative to avoidance responding to obtain a new signal which only differs in color. (*A*) Training data depicting avoidance responses to the CSs+ and CSs−. Each block contains three trials. Participants made more responses to the CSs+ across blocks compared to the CSs−. (*B*) Test data comparing avoidance responses to Gabor that was followed by the trained signal versus responses to the new signal. Participants made a similar number of avoidance responses to obtain the trained safety signal and the new signal. Error bars depict standard error of the mean.

During training, participants (*n* = 55) made more avoidance responses to the CSs+ than to the CSs−, and this difference became larger as training progressed (Fig. 2A). This was supported by a 2 CS (CSs+ vs. CSs−) × 15 (Trials) repeated-measures ANOVA, which revealed an effect of CS, *F*_(1,54)_ = 7.62, *P* = 0.008, $\eta_{p}^{2}$ = 0.124, a main effect of trial, *F*_(7.10,383.83)_ = 5.18, *P* < 0.001, $\eta_{p}^{2}$ = 0.088, and a significant interaction, *F*_(8.70,470)_ = 3.30, *P* < 0.001, $\eta_{p}^{2}$ = 0.058. We also assessed the number of safety signals participants produced in the training phase by conducting a one-way ANOVA with Blocks (1–5; see Table 1 for descriptives). As the blocks progressed, the number of safety signals produced increased, *F*_(3.54,191.13)_ = 7.56, *P* < 0.001, $\eta_{p}^{2}$ = 0.123.

During the test phase (Fig. 2B), participants responded similarly to the Gabor that was followed by the trained safety signal and that which was followed by the new signal. These impressions were confirmed by a 2 (Signal: Trained Signal vs. New Signal) and Trials (1–8) repeated-measures ANOVA, that revealed a main effect of trials—as test trials progressed responding decreased, *F*_(3.26,176.53)_ = 6.60, *P* = < 0.01, $\eta_{p}^{2}$ = 0.109. There was, however, no effect of signal, revealing that participants made the same number of avoidance responses to the Gabor that was followed by the trained safety signal and that was followed by the new signal, *F*_(1,54)_ = 0.003, *P* = 0.958. Furthermore, there was no interaction between the type of signal and trials, *F*_(5.9,318.58)_ = 1.55, *P* = 0.16. Because responding to either Gabor patches during the test was followed by a signal, we also assessed the overall number of signals participants produced in the test phase with a paired *t*-test. There was no difference in the overall number of trained safety signals produced (*M* = 5.71, SD = 2.95), and the overall number of new safety signals produced (*M* = 5.35, SD = 2.93), *t*_(54)_ = 1.018, *P* = 0.31. After the behavioral test, we assessed the expectancy data for the CS+ and the CS− and found that participants rated the CS+ as more likely to be followed by the aversive stimulus than the CS−. A paired *t*-test revealed there was a significant difference between the CS+ (*M* = 6.24, SD = 2.63) and the CS− (*M* = 3.36, SD = 2.54); *t*_(54)_ = 6.34, *P* < 0.001, Cohen's *d* = 0.856.

A possible reason for the participants responding similarly to both signals is that during training, the discrimination between CS+ and CS− was not very strong, despite it being significant, and this somehow transferred to the new Gabors presented during the test. To assess this possibility, we calculated a discrimination score during training (CS+/CS−) and assessed the correlation of this with the responses to obtain the trained signal/responses to obtain the new signal. This correlation was not significant, *r*_(53)_ = 0.122; *P* = 0.376. A second possibility is that responding was similar because of generalization. Because the signals used in this experiment only differed in one dimension (color), it could be possible that participants responded similarly to the two Gabor patches because they generalized from the trained safety signal to the new (control) signal at the test. This speculation is supported by findings from Dinsmoor and Sears (1973), who observed that during the test, pigeons avoided response-produced auditory signals in a way that resulted in a generalization gradient. Avoidance responding was highest when the response-produced safety signal was the same as that presented during training (1000 Hz), somewhat lower to stimuli of similar frequency (500 and 2000 Hz) in some subjects, and much lower to stimuli that were different from the trained safety signal (200 and 4000 Hz). As the response-produced signal during the test was made more different from the trained safety signal, responses decreased. If this were the case, we expected to observe different response rates to Gabors followed by the trained safety signal versus a new safety signal, provided that the new signal at the test was sufficiently different from that presented during training. Experiment 3 was designed to test this hypothesis.

### Experiment 3: Participants made more avoidance responses to the stimulus that was followed by the trained safety signal compared to a new dissimilar signal

Experiment 3 was similar to Experiment 2 in all aspects, except that the trained safety signal and the new (control) signal presented at the test were made more dissimilar. That is, the signals were different in color (green vs. pink) and shape (square vs. triangle). In this experiment, we assess evidence that safety signals have reinforcing properties at the test when comparing avoidance responding to a Gabor that produced the trained signal (e.g., green triangle) to a Gabor that is followed by a new dissimilar signal (e.g., pink square).

**Figure 3.**
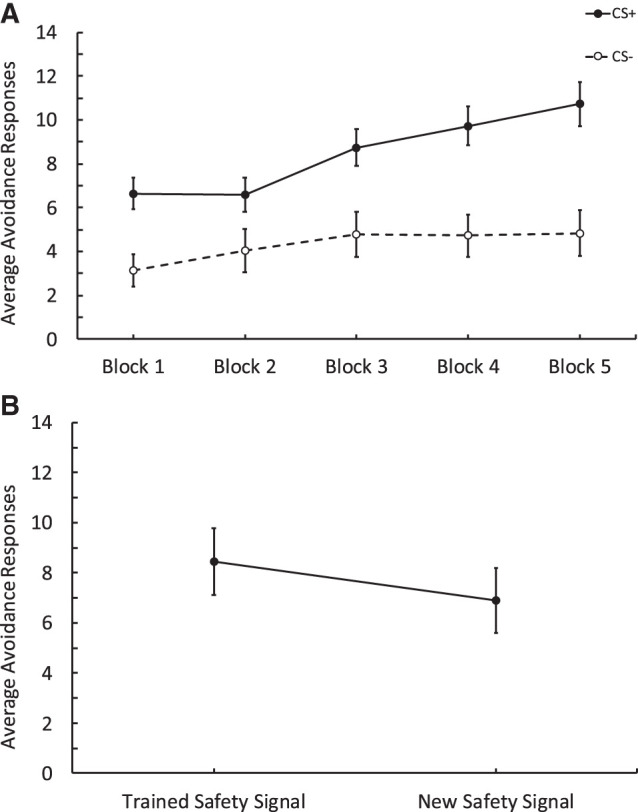
Results from Experiment 3 comparing avoidance responses to obtain a response-produced safety signal relative to a new dissimilar signal. (*A*) Training data depicting avoidance responses to the CSs+ and CSs−. Each block contains three trials. Participants made more responses to the CSs+ across blocks compared to the CSs−. (*B*) Test data showing avoidance responses to the Gabor that was followed by the trained safety signal versus responses to a Gabor that was followed by a new dissimilar signal. Participants made more responses to obtain the trained safety signal relative to the new dissimilar signal. Error bars depict standard error of the mean.

Figure 3A depicts the training data (*n* = 74). We conducted a 2 CS (CSs+ vs. CSs–) and Trials (1–15) repeated-measures ANOVA. In line with what we observed in previous experiments, participants responded during training more to the CSs+ compared to CSs−, and this changed throughout training, as evidenced by a significant interaction between CS and trials, *F*_(7.90,577.19)_ = 5.32, *P* < 0.001, $\eta_{p}^{2}$ = 0.068. There were also main effects of CS, *F*_(1,73)_ = 31.73, *P* < 0.001, $\eta_{p}^{2}$^=^0.303 and trials, in that as trials increased, responding also increased: *F*_(7.75,565.81)_ = 9.02, *P* < 0.001, $\eta_{p}^{2}$ = 0.11. Like previous experiments, we also assessed the number of safety signals participants produced during the training phase by conducting a one-way ANOVA (Blocks: 1–5; see Table 1 for descriptives). As training blocks progressed, the number of safety signals participants produced increased: *F*_(3.30,240.99)_ = 12.27, *P* < 0.001, $\eta_{p}^{2}$ = 0.144.

Critically, during the test phase (see Fig. 3B), participants responded to the Gabor that was followed by the trained safety signal more than that followed by the new dissimilar signal. A repeated-measures ANOVA 2 (Signal: Trained Signal vs. New Signal) and Trials (1–8) conducted on avoidance responses during the test revealed an effect of the signal, showing that participants made more avoidance responses to the Gabor which was followed by the trained safety signal and relative to that followed by the new signal, *F*_(1,73)_ = 4.90, *P* = 0.029, $\eta_{p}^{2}$ = 0.064. The ANOVA also revealed an effect of trials (as the test trials progressed, responding decreased), *F*_(3.46,252.67)_ = 4.76, *P* = 0.002, $\eta_{p}^{2}$ = 0.061, but there was no interaction between these factors, *F*_(3.90,284.89)_ = 0.72, *P* = 0.574.

Similarly, a paired *t*-test was conducted to assess the number of trained safety signals produced compared to the new safety signal. It was found that the number of trained safety signals produced (*M* = 4.3, SD = 3.13) was higher than the number of new safety signals produced (*M* = 3.61, SD = 3.01), *t*_(73)_ = 3.60, *P* < 0.001, Cohen's *d* = 0.419. Finally, we assessed the expectancy data for the CS+ and the CS− and found that participants rated the CS+ as more likely to be followed by the aversive stimulus than the CS−. A paired *t*-test revealed there were significant differences in expectancy scores between the CS+ (*M* = 6.72, SD = 2.67) and the CS− (*M* = 2.49, SD = 2.26); *t*_(73)_ = 10.69, *P* < 0.001, Cohen's *d* = 1.24.

Collectively, Experiments 1 and 3 provide convincing evidence that response-produced safety signals reinforce avoidance behavior. However, the experiments were conducted online, and under these conditions, there is little experimental control over the situation. Experiment 4 was run to address this concern, by attempting to replicate Experiment 1, but recruiting participants from the School of Psychology and using a loud tone as the aversive stimulation.

### Experiment 4: Participants made more avoidance responses to stimuli that produced a trained safety signal compared to stimuli that produced nothing

**Figure 4.**
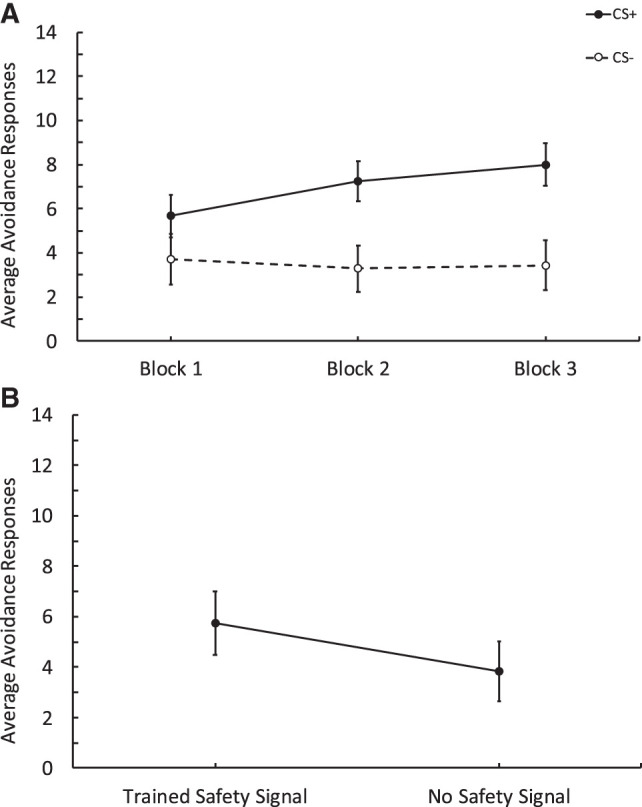
Results from Experiment 4 conducted in the laboratory comparing avoidance responses to obtain a response-produced safety signal relative to no signal. (*A*) Training data depicting avoidance responses to the CSs+ and CSs−. Each block contains three trials. Participants made more responses to the CSs+ across blocks compared to the CSs−. (*B*) Test data showing avoidance responses to the Gabor that was followed by the trained safety signal versus responses to a Gabor that was followed by no signal. Participants made more responses to the Gabor followed by the trained safety signal than to the Gabor that was followed by no signal. Error bars depict the standard error of the mean.

Experiment 4 was the same as Experiment 1 except it was conducted in the laboratory, so the aversive stimulus was a loud tone (95 dB), and there were only three blocks of training during the instrumental phase as opposed to five blocks. Everything else remained the same. We conducted a 2 CS (CSs+ vs. CSs–) and Trials (1–9). As observed in the three previous experiments, during training participants responded more to the CSs+ than the CSs−, and this changed as the blocks progressed (see Fig. 4A) as revealed by a significant CS by trial interaction, *F*_(5.4,216.04)_ = 2.81, *P* = 0.015, $\eta_{p}^{2}$ = 0.066. There was also a main effect of CS, *F*_(1,40)_ = 16.98, *P* < 0.001, $\eta_{p}^{2}$ = 0.298, but no effect of trials, *F*_(4.69,187.78)_ = 2.01, *P* = 0.083, $\eta_{p}^{2}$ = 0.048. Furthermore, we assessed the number of safety signals produced and observed that these increased throughout the three training blocks (see Table 1 for description), *F*_(1.64,65.66)_ = 20.54, *P* < 0.001, $\eta_{p}^{2}$ = 0.339.

Critically, during the test phase participants made more avoidance responses to the Gabor that was followed by the trained safety signal (see Fig. 4B). A 2 (Signal: Trained Signal vs. No Signal) and Trials (1–8) repeated-measures ANOVA revealed a main effect of signal, *F*_(1,40)_ = 6.20, *P* = 0.017, $\eta_{p}^{2}$ = 0.134. There was also a significant effect of trials in that as they progressed responding decreased, *F*_(3.06,122.56)_ = 5.64, *P* = 0.001, $\eta_{p}^{2}$ = 0.124; however, there was no interaction between these factors, *F*_(4.39,175.79)_, 0.55, *P* = 0.71, $\eta_{p}^{2}$ = 0.014. After the behavioral test, we assessed the expectancy data for the CS+ and the CS− and found that participants rated the CS+ as more likely to be followed by the aversive stimulus than the CS−. A paired *t*-test revealed there was a significant difference in expectancy scores between the CS+ (*M* = 7.12, SD = 2.08) and the CS− (*M* = 2.59, SD = 2.12); *t*_(40)_ = 9.07, *P* < 0.001, Cohen's *d* = 1.41.

Experiment 4 revealed that, in line with the findings from Experiment 1, participants made more avoidance responses to the ambiguous Gabor patch that produced the safety signal compared to the one which did not result in any signal. Thus, this experiment conducted in person confirms previous findings and reinforces the finding that response-produced safety signals reinforce avoidance behavior in humans. This shows that the findings from Experiment 1 are reliable and can be replicated in the laboratory with a different aversive stimulus (loud tone) which strengthens these overall findings. In summary, these experiments show that safety signals have reinforcing properties in human avoidance behavior. We observed this in Experiments 1 and 4 when we tested avoidance responses to a novel stimulus which was followed by the trained safety signal in comparison to another stimulus that was followed by nothing. Experiments 2 and 3 further clarified the role of the safety signal by revealing differential responses to stimuli when followed by a trained safety signal relative to a novel stimulus followed by a dissimilar (Experiment 3) but not similar (Experiment 2) control signal. We wanted to further test the reinforcing properties of the safety signal by investigating if they transfer to a new response. Therefore, in Experiment 5, we set out to test this by changing the response in the test phase of the experiment.

### Experiment 5: Participants display a preference to respond to a stimulus that is followed by the trained safety signal compared to a stimulus that is followed by a dissimilar new signal

Experiment 5 was similar to Experiment 3 except for a minor change during training (see Materials and Methods) and two notable differences. During the test phase, the two novel Gabors were used (45° and 135°), as in previous experiments. However, they were now both presented on the screen at the same time, so participants had the choice between the stimuli. Furthermore, we also changed the response required from the participants. Instead of pressing the spacebar they now had to direct the mouse pointer and click on the Gabor image. If they pressed more to one of the Gabors, the stimulus (safety signal or control) that the Gabor produced was presented. If they did not press either, then nothing was presented and if they pressed the same number of times to each Gabor, the stimulus produced by the Gabor they pressed first was presented. The timings of the stimuli and all other aspects of the task were the same as Experiment 3. If safety signals reinforce avoidance behavior, we expected participants to respond (click) more on the Gabor patch that produced the safety signal when given a choice between the two despite both Gabor's being novel and never paired with the aversive outcome.

**Figure 5.**
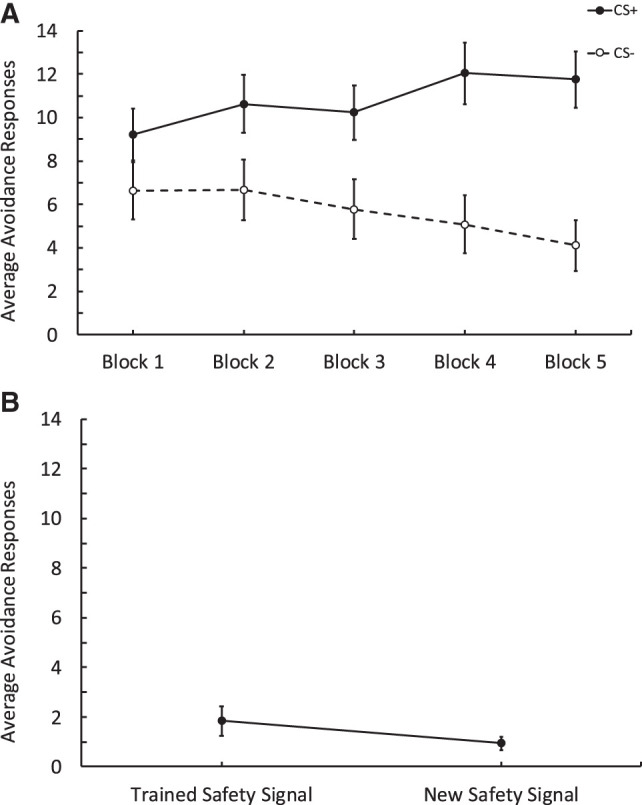
Results from Experiment 5 comparing avoidance responses to obtain a response-produced safety signal relative to a new dissimilar signal. (*A*) Training data depicting avoidance responses to the CSs+ and CSs−. Each block contains three trials. Participants made more responses to the CSs+ across blocks compared to the CSs−. (*B*) Test data showing avoidance responses (new response compared to training) to the Gabor that was followed by the trained safety signal versus responses to a Gabor that was followed by a new dissimilar signal. Participants made more responses to obtain the trained safety signal relative to the new dissimilar signal. Error bars depict standard error of the mean.

Figure 5A shows that during training, as trials progressed participants (*n* = 54) responded more to the CSs+ than the CSs−. To assess this, we measured the rate of responding for each trial during the training phase. A repeated-measures ANOVA with CS (CSs+ vs. CSs−) and trials (Trials 1–15) revealed a main effect of CS, *F*_(1,53)_ = 34.95, *P* < 0.001, $\eta_{p}^{2}$ = 0.39, trial *F*_(7.47,395.89)_ = 2.48, *P* = 0.014, $\eta_{p}^{2}$ = 0.045, and a significant interaction, *F*_(8.79,466.16)_ = 5.02, *P* < 0.001, $\eta_{p}^{2}$ = 0.087. We also analyzed the number of safety signals that participants experienced during the training phase. As training blocks progressed, the number of safety signals received increased (see Table 1 for descriptives). A one-way ANOVA revealed a significant effect of blocks, *F*_(3.38,179.44)_ = 3.17, *P* = 0.021, $\eta_{p}^{2}$ = 0.057.

During the test, we found a marginal difference between responding to the Gabor followed by the trained signal and the Gabor followed by the new signal. A repeated-measures ANOVA with Signal (Trained Signal, New Signal) and Trials (1–8) revealed there was a marginal effect of Signal, *F*_(1,53)_ = 3.29, *P* = 0.075, $\eta_{p}^{2}$ = 0.058. Furthermore, there was no effect of trial, *F*_(3.52,186.68)_ = 0.707, *P* = 0.57, and there was no interaction between signal and trials, *F*_(3.76,199.59)_ = 0.56, *P* = 0.68. Visual observation of the test results revealed that responding decreased numerically after Trial 4; therefore, we analyzed the first four test trials alone. Participants made more avoidance responses to the Gabor stimulus that was followed by the safety signal compared to the one that was not (see Fig. 5B). A repeated-measures ANOVA with Signal (Trained Signal vs. New Signal) and Trials (1–4) compared avoidance responses to the Gabor followed by the safety signal versus the one that was followed by a new signal. There was a main effect of signal, *F*_(1,53)_ = 5.48, *P* = 0.023, $\eta_{p}^{2}$ = 0.094, revealing more avoidance responses to the Gabor followed by the safety signal, there was no effect of trial, *F*_(1.86,99.01)_ = 0.256, *P* = 0.759, there was also no interaction between these trials and signal, *F*_(2.27,120.39)_ = 0.502, *P* = 0.630. A paired *t*-test was conducted to assess the number of trained and new safety signals produced during test. It was found that that the trained safety signal was produced more (*M* = 2.91, SD = 2.69) than the new safety signal (*M* = 1.54, SD = 2.07), *t*_(53)_ = 2.89, *P* = 0.006, Cohen's *d* = 0.394.

Finally, after the choice tests we assessed the participants' expectancy ratings for the CS+, CS−, and the two novel test stimuli. We found that participants rated the CS+ (*M* = 6.8, SD = 2.3) higher than the CS−, (*M* = 3.19, SD = 2.23); *t*_(53)_ = 8.30, *P* < 0.001, Cohen's *d* = 1.13. Finally, there were no differences in expectancy ratings to the test CS paired with the training signal and the test CS paired with the new signal; *t*_(53)_ = 1.49, *P* = 0.141, although there was a trend toward more expectancy ratings to the novel CS that was followed by the safety signal (*M* = 5.41, SD = 2.17) relative to the one which was followed by the new signal (*M* = 5, SD = 2.29).

Experiment 5 revealed that, when given the choice between two novel stimuli, participants emitted more avoidance responses to an ambiguous Gabor patch which resulted in the trained safety signal relative to one which resulted in a new signal. It should be noted that during the test, the number of responses (i.e., mouse clicks) was appreciably lower than the responses observed at the test in previous experiments. This is not surprising given that there is an expected decrement that results from the transfer from one response during training (space bar presses) to a different response during the test (mouse clicks). Nevertheless, there was a significant difference (during the first four test trials) and hence these findings suggest that the safety signal reinforced avoidance behavior, even when tested with a new response. This builds on the previous experiments and showcases that when the response changes, participants still have a preference for the trained safety signal as opposed to the new signal suggesting the reinforcing properties can transfer not only to new stimuli, but also to new responses.

## Discussion

This study assessed the reinforcing properties of safety signals in a human instrumental avoidance paradigm. We observed that safety signals can reinforce avoidance behavior elicited by stimuli without a history of avoidance reinforcement, and we also found that the reinforcing properties of safety signals can easily generalize to other stimuli. Experiments 1 and 4 revealed that trained safety signals reinforced behavior when compared with a control condition that was not followed by a signal. Because the findings in Experiments 1 and 4 can be explained by sensory reinforcement, Experiments 2, 3, and 5 used a new signal as a control for the trained safety signal. In Experiment 2, when the control signal was similar to the trained safety signal, no differences in avoidance responses (nor in terms of number of signals earned) between trained and similar control signals were observed at the test. It was only when the trained safety signal differed from a new signal in two dimensions (color and shape) that higher avoidance responses for the trained safety signal were observed. To our knowledge, this is the first quantitative demonstration of safety signals reinforcing avoidance behavior in humans, despite a recent resurgence of research interest on the topic (Vervliet and Indekeu 2015; Gillan et al. 2016; Vervliet et al. 2017; Urcelay and Prével 2019).

There have been several experiments conducted in animals which support the notion that safety signals reinforce avoidance behavior. Previous work in rats has shown that when given the choice between a lever that produces a safety signal and one that does not, rats preferred the one with the signal, despite both levers resulting in successful avoidance of a shock (Fernando et al. 2014a). This finding was further confirmed by a choice test on extinction, which revealed more avoidance responses to a lever which was followed by the safety signal relative to a control lever which was not. Additional studies with selective infusions of dopaminergic agonists and antagonists in subregions of the ventral striatum documented that the reinforcement of safety signals is a dopamine-dependent phenomenon (Fernando et al. 2014b). Furthermore, work by Dinsmoor and Sears (1973) showed that pigeons pressed a pedal to produce a tone trained as a safety signal compared with no tone, or a tone of a different frequency. In our Experiment 2, participants responded at equal rates to the trained safety signal and a control signal that was somewhat similar to the trained safety signal. It was only when the control signal was sufficiently different from the trained safety signal that we observed a difference in Experiments 3 and 5. This could be seen as similar to the pattern of results observed by Dinsmoor and Sears (1973), although it would be interesting to test participants with a wider range of new signals—that is, future research may want to better assess the generalization of the properties of the safety signal. Furthermore, in Experiment 5, we observed that safety signals can reinforce a new response which has been proposed as the ultimate way to determine the reinforcing properties of a conditioned stimulus (Mackintosh 1974).

The current findings in humans add to the existing literature suggesting similar motivational processes in positive reinforcement (for rewarding outcomes) and negative reinforcement (for aversive outcomes) that have been documented at neural (Seymour et al. 2005; Leknes et al. 2011) and behavioral levels (Leng et al. 2023). Overall, these results are consistent with an architecture that assumes two separate motivational systems that interact by inhibiting each other (Konorski 1967; Dickinson and Dearing 1979). According to this model, stimulation of the appetitive system results in the expectation of an appetitive outcome (hope) and also in inhibition of the aversive system. Similarly, stimulation of the aversive system results in the expectation of an aversive outcome (fear) and inhibition of the appetitive system. On the contrary, inhibition of the aversive system by signals associated with the absence of aversive reinforcement (safety signals), disinhibits the appetitive system giving rise to the positive feeling of relief. Thus, the reciprocity between appetitive and aversive systems, which has been demonstrated with Pavlovian procedures (Dickinson and Dearing 1979), is here substantiated with instrumental avoidance responses in humans. A recent computational instantiation of a dual-process theory of instrumental behavior assumes that instrumental behavior is controlled by a goal-directed component and a habitual component (Perez and Dickinson 2024). According to this account, safety signals reinforce the habitual component of avoidance behavior by strengthening the connection between environmental stimuli and avoidance responses, as has been observed in experiments in rodents (Fernando et al. 2014a).

To strengthen the findings of this study, further research could assess physiological responses with the use of the in-person procedure. It would also allow for physiological measures such as skin conductance to be taken which would further add to the behavioral (i.e., avoidance) measures we have obtained from the current study. It would also be interesting to gather participant subjective ratings, such as those used by Vervliet et al. (2017), for example, by asking participants how much relief they felt after avoiding. Having a combination of the physiological, behavioral, and self-report measures may allow a more complete understanding of avoidance behavior.

Furthermore, a potential limitation of the first four experiments was that during the expectancy test the two test stimuli were not included. Without this, it opens the opportunity for the suggestion that participants classified the test stimuli as threatening (belonging to the CS+ stimuli set), depending on whether the safety signal was presented or not at the test. This logic could potentially explain why participants responded to the stimuli that produced the trained safety signal more than the stimuli that were used as control. However, in Experiment 5, this was investigated as we assessed expectancy ratings to the test stimuli, and participants rated the two test stimuli similarly: notably both test stimuli were rated descriptively lower than the CS+. This means that perhaps the current findings can only be observed in situations in which there is high ambiguity, so further research using free-operant procedures (as was used in the study by Fernando et al. 2014a,b) is needed to clarify this possibility.

Finally, this study was conducted with healthy individuals so it would be interesting to assess a clinical population of people with diagnosed anxiety disorders to see if their behavior follows the same pattern as those found in this study, and critically if anxiety mediates the reinforcing properties of safety signals as was recently documented with subjective measures (De Kleine et al. 2023). In our experiments (see Supplemental Material), we recruited healthy participants and asked them to complete the STAI. In each experiment, participants were split into high and low anxiety based on a median split of scores. Although we did not see any relevant interaction between safety signal responding and anxiety group, we did see across all experiments that participants high in anxiety tended to press more both during avoidance training and test, consistent with the notion that high avoidance responding is a hallmark of anxiety (see Supplemental Figs. S2–S7).

In summary, we observed that safety signals can reinforce instrumental avoidance behavior in human participants. The role of safety signals has been highlighted as important in the development and maintenance of anxiety disorders (Lohr et al. 2007), and there is currently interest in how inhibition interacts with fear and avoidance to better understand anxiety disorders (Sangha et al. 2020; Cassaday et al. 2023). Hence, understanding the reinforcing properties and their boundaries is of interest to the field. The current demonstration in humans is a first step into understanding safety signals and their reinforcement of avoidance behavior. These findings in humans will allow us to begin understanding the role safety signals play in maladaptive avoidance.

## Materials and Methods

### Participants

All four online experiments recruited participants via Prolific with the selection criteria being the same for all experiments. The participants were from unique participant pools with one of the screening criteria being that they had not taken part in any of the previous experiments on safety signals. Participants had to be between 18 and 60 years old, have normal or corrected vision, no color-blindness and could speak English fluently. We had a training criterion which participants had to pass. This was included so that the participants understood the task and what was expected (no safety signals were shown in this phase). Participants had to respond more to the CS+ compared to the CS− for two consecutive blocks. If they did this they moved on to the experiment and if they did not pass then the experiment went straight to the end screen. The first replication in Experiment 1 which included 50 participants, revealed that the CS+ versus CS− discrimination learning during training with these stimuli achieved a power = 0.91, so in each experiment, we aimed to recruit 50 participants that completed the task. However, after the data were collected (Experiments 1–3), we noticed that some participants did not earn any safety signals during training. Because the objective of this study was to assess the reinforcing properties of safety signals, we excluded participants that during training did not experience any safety signals. This decision was made because we were interested in assessing the reinforcing properties of the safety signals; therefore, we needed participants to experience them during training in order to draw conclusions about the effect of a trained safety signal has on avoidance behavior. Therefore, each of the online experiments was run a second time until 50 additional participants completed the task. In hindsight, we should have preregistered these replications. Of note, if we run all the reported tests but including all 100 participants that completed the task in each experiment, the results are exactly the same as those reported after these exclusions. In no case does replication interact with any of the results reported. The experiment in the laboratory was run based on the power analyses mentioned above and data collection was stopped when 50 participants had completed the task. We applied to the ethics committee in the School of Psychology at the University of Nottingham and were granted clearance to run the experiments in this paper. The ethics code for the project is (S1402). All the data collected can be found at: https://osf.io/xazet/.

In Experiment 1, 131 participants were recruited; however, 18 did not complete the full experiment, and 13 people did not pass the pretraining criteria (see below). The final sample was 100 participants which consisted of 49 males and 51 females. The ages ranged from 18 to 60 years old (*M* = 30.66, SD = 8.52). In Experiment 2, 143 participants were recruited; however, 28 people did not complete the full experiment, and a further 15 people did not pass the pretraining criteria. The final sample was 100 with 49 females and 51 males. The ages ranged from 18 to 40 years old (*M* = 29.4, SD = 5.73). In Experiment 3, we recruited 129 participants. However, 16 did not complete the experiment and were excluded, and 13 did not pass the training criteria so were also excluded. Therefore, the final sample had 100 participants, which consisted of 45 females, one nonbinary person, and 54 males. The ages ranged from 20 to 49 years old (*M* = 30.57, SD = 6.32). In Experiment 4, 51 people were recruited, one person did not pass the pretraining criteria, so the final sample was 50. The sample consisted of 12 males and 38 females. The ages ranged from 18 to 28 years old (*M* = 19.32, SD = 2.025). Experiment 5 was preregistered (https://doi.org/10.17605/OSF.IO/ZXAES), and on the basis of Experiment 3, we conducted a power analysis which revealed that we needed 49 participants to achieve 0.95 power to detect a difference at the test. On the basis of the participant attrition observed in previous online experiments, we recruited 73 participants, which were recruited via Prolific. The sample consisted of 47 females, 25 males, and one nonbinary person. The ages ranged from 18 to 65 (*M* = 34.8, SD = 10.87).

After applying the criterion that participants had to earn at least one safety signal during training, Experiment 1 had a final sample of 72 participants. There were 37 males and 35 females. The ages ranged from 18 to 60 (*M* = 30.96, SD = 8.55). In Experiment 2, there were 55 participants with 26 females and 29 males. The ages ranged from 18 to 40 (*M* = 29.6, SD = 6.43). Experiment 3 had 74 participants: 34 females and 40 males, and the ages ranged from 21 to 49 (*M* = 31.12, SD = 6.49). In Experiment 4, there were 41 participants: 11 males and 30 females. The ages ranged from 18 to 28 (*M* = 19.46, SD = 2.203). In Experiment 5, there were 54 participants, and ages ranged from 18 to 65 (*M* = 35.13, SD = 11.05), with 35 females, 18 males, and one nonbinary person.

### Materials

The aversive outcome in Experiments 1–3 and 5 was an aversive image. These images were selected from the international affective picture system (IAPS). The images chosen were aversive but not traumatic and all images had similar arousal and aversive ratings (see Supplemental Table S1). At the beginning of the experiment, participants had to rank a series of six images from least to most aversive. The image that was selected as the second most aversive was used as the outcome image during the experiment. The images included a spider, snake, a dirty toilet, a cockroach on a pizza, a surgery, and a person vomiting (see Supplemental Material for the valance and arousal ratings for each image).

The aversive outcome in Experiment 4 was a loud tone. The tone was delivered via headphones which were calibrated to ensure the tone was 95 dB. Participants were aware before taking part in the experiment that an aversive sound would be used and that if they found it too loud, they could request for the volume to be reduced, however, none of the participants requested this.

Participants completed a short questionnaire to assess their anxiety levels. The first was Spielberger's State-Trait Anxiety Inventory (Spielberger and Craighead 2010), which contains 40 questions, all of which have a 4-point Likert scale from 1 to 4 (with 1 being not at all and 4 being very much so). The questions assess participants’ current feelings, for example, “I feel calm” and “I feel frightened.”

There were two fractal images used during a pretraining phase for all experiments (see Supplemental Fig. S1). These images were abstract fractals of different colors. One was paired with the aversive outcome, and one was not, and participants had to press the space bar to avoid seeing the aversive outcome.

**Figure 6.**
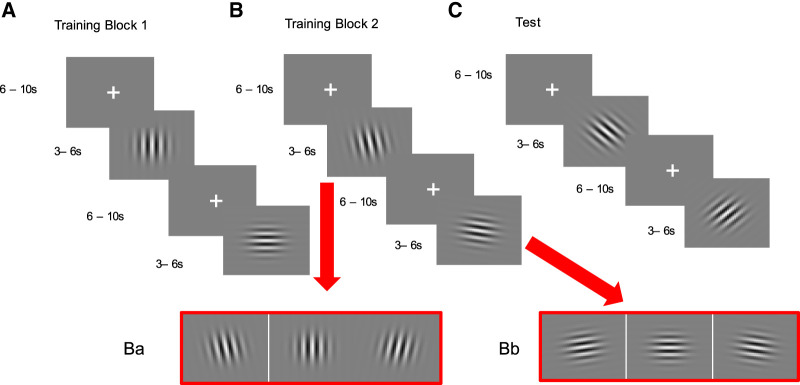
There are two phases within these experiments: training and test. Training was first started with an easy Block 1 (*A*) which was followed by more difficult blocks (Blocks 2–5 in Experiments 1, 3, and 5, and Blocks 2–3 in Experiment 4). This was followed by a test with novel stimuli (*C*), one of which was followed by the trained safety signal and the other served as a control. (*A*) Training Block 1. Participants were shown either a horizontal or vertical Gabor patch. One was paired with an aversive image or a loud noise (CS+), one was not (CS−), and this was counterbalanced. (*B*) Training Blocks 2–5, in which more stimuli were incorporated. Four new stimuli were added (*Ba* and *Bb*). Each of the new Gabor stimuli were 10° apart in orientation from the stimuli used in Training Block 1, except Experiment 5 in which Block 2 had 5° of difference. This made the “vertical” versus “horizontal” discrimination more difficult, but also facilitated the transfer of responding during the test to the new stimuli. (*C*) The Test Phase participants were shown new stimuli. These Gabor patches were in between the horizontal and vertical Gabor stimuli (135° and 45°). One of these produced the trained safety signal and the other one was the control.

There were eight Gabor images used as CSs in this experiment (see Fig. 6A–C). They all had the same spatial frequency (10c/deg) and size but differed in terms of the orientation. There was a vertical set (Fig. 6Ba) and horizontal set (Fig. 6Bb) used during training, both of which included three stimuli. These sets were used as the CS+ and the CS− (these were counterbalanced between participants). The horizontal set included Gabor patches at 0°, 10°, and 350° and the vertical set included stimuli at 90°, 80°, and 100°. During the test (Fig. 6C), two other Gabors were used (45° and 315° angles).

**Figure 7.**
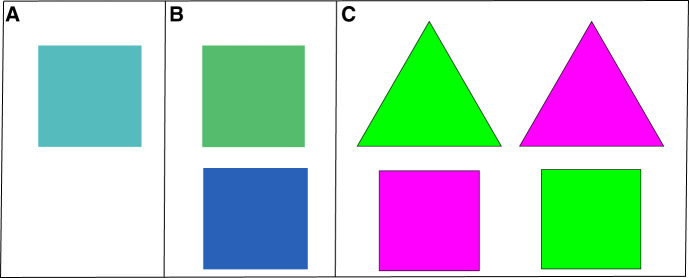
This figure shows the different stimuli used as safety signals (and control) for each experiment. (*A*) The stimulus used as safety signal Experiments 1 and 4. All participants were trained with the same safety signal (aqua square), and the control during the test phase was no safety signal. (*B*) The stimuli used in Experiment 2. Participants were trained with either green or blue squares. During the test, the control was the square that they had not been trained with. (*C*) The stimuli used in Experiment 3. Participants were trained with either a square or triangle and this was either pink or green. During the test, the control was the opposite shape and color. For example, if a participant was trained with a green triangle, then the control during the test was a pink square. Stimuli were counterbalanced.

The safety signals differed for each experiment (see Fig. 7). In Experiment 1, the trained safety signal was an aqua color that was selected from the middle of the blue–green color continuum (Fig. 7A). This was presented as a square (in the center of the screen while the background was gray). In Experiment 2, the trained safety signal was either dark blue or dark green, counterbalanced across participants (Fig. 7B). In Experiment 3, the safety signals were either a triangle or a square and they could be either pink or green (Fig. 7C).

### Procedure

Experiments 1–3 and 5 took place online. After filling out a consent form, participants filled out the anxiety scale (STAI). Following this, the participants ranked each of the six aversive images which served as the aversive outcome. They ranked them from least to most aversive and the image selected as the second most aversive was used as the aversive outcome for the remainder of the experiment. This experiment involved four phases. These were pretraining, training, test, and expectancy. Experiment 4 was run in the laboratory and did not include the ranking of aversive images. In addition, only three blocks of training were administered, because participants in the laboratory learn much better and do not need so much training to reliably discriminate between CSs+ and CSs− during training.

### Pretraining

The first phase was the pretraining phase. During this phase, participants were presented with two fractal images. These were square images (they were 50% of the screen height and presented in the middle of the screen). The background was gray. One fractal served as the CS+ and was followed by the aversive outcome (aversive image). The other was the CS− and was followed by nothing. Participants were told that they could avoid the aversive image by pressing the space bar on the keyboard. In the CS+ trials, the US could appear on seconds 4, 5, or 6 of this trial. If an avoidance response was performed 1 sec before the US was scheduled to appear, avoidance was successful and the trial ended. If the avoidance response was unsuccessful, then the aversive outcome was played for 1 sec. Each trial was variable in length due to the variability in CS duration (3–6 sec), and the intertrial interval was variable (6–10 sec) with an average of 6 sec. During the ITI, a white fixation cross was presented in the center of the screen. There were two presentations of the CS+ and CS− per block. The participants had to meet a criterion before they were able to move on. For them to move on to the next phase, they had to respond more to the CS+ than the CS− for two consecutive blocks. Pretraining had a maximum of 10 blocks and was performed to make sure all participants understood the nature of the avoidance task.

### Training

The second phase of the experiment was the training phase which was similar to the pretraining phase, except that it used the Gabor stimuli instead of fractals (see Fig. 6A). This phase was split into two parts. During Training Block 1, participants were shown two Gabor patches. One was horizontal and the other was vertical. One of these was paired with the aversive image (CS+) and the other was not (CS−). The aversive outcome could be avoided if the participants pressed the spacebar 1 sec before the aversive outcome would appear. If avoidance was successful, the CS terminated, the aversive image was not shown and instead the trained safety signal was presented for 3 sec. If unsuccessful, the CS was terminated, and the aversive image (US) was shown for 1 sec. The presentation of the aversive image was variable and could occur on seconds 4, 5, or 6 of the 6 sec trial. Therefore, the response windows for successful avoidance were seconds 3, 4, and 5. Each of the Gabor patches was presented three times each.

Training Blocks 2–5 were the same as Training Block 1 (three trials) except that new stimuli were added and thus made more difficult (see Fig. 6B). Participants were presented with four new Gabor patches. Two of these new stimuli differed 10° from the horizontal stimuli and two differed 10° from the vertical stimuli. They therefore made two sets of stimuli: a horizontal set and a vertical set. If the CS+ was 90°, then the CSs+ during Blocks 2–5 were 80°, 90° or 100°, and the CSs− were 350°, 0°, and 10°. There were four difficult blocks and each of the six stimuli was shown once per block so there were 24 trials in total for Experiments 1–3. In Experiment 4, everything was the same except there was a reduced amount of difficult blocks (two blocks). In total, participants experienced 15 CS+ and 15 CS− trials during Experiments 1–3. In Experiment 4, participants experienced a total of nine CS+ trials and nine CS− trials. Experiment 5 had the same Training in Block 1, but Block 2 introduced new stimuli 5° away from those in Block 1 (85°, 90°, 95°, and 355°, 0° and 5°). Blocks 3–5 were similar to Experiments 1–4.

### Test

#### Experiments 1–4

The next phase of the experiment was the test phase which occurred during extinction, meaning that the aversive outcome was no longer presented. However, participants were not explicitly informed of this and moved straight from the instrumental phase into the test phase with no instructions in between. They were presented with two new Gabor gratings which were 45° and 135°. In the first four experiments, one of these Gabor patches produced the trained safety signal if the participants made an avoidance response. The participants only needed to respond 1 sec after the CS first appeared on the screen in order for the safety signal to appear at the end of the Gabor presentation.

In Experiments 1 and 4, the alternative (control) Gabor patch produced nothing if the participant responded. In Experiment 2, the alternative Gabor patch produced a signal that was the opposite color to the trained safety signal, but of the same shape. That is, if the trained signal was blue then the test control was green. In Experiment 3, the alternative Gabor patch produced a signal that was of a different color and shape. For example, if the trained safety signal was a green triangle, then the test control was a pink square. The test included eight trials of each degree angle gratings (16 in total).

### Test

#### Experiment 5

In Experiment 5, the test was different as both stimuli were shown on screen at the same time. The side the two Gabors were shown on was randomized at the start of the test but then they remained on the side throughout the test phase. The Gabor stimuli were presented on each side of the screen, both an equal distance from the middle and the edge of the screen. The Gabors were on the screen for 6 sec and then there was a 6–10 sec ITI. There were eight test trials in total. The trained safety signal and the new signal were the same as the ones used in Experiment 3. Also, the response had now changed in the test phase and the participants had to click on the Gabor patches. Whichever Gabor they clicked would then show the safety signal (or alternative new signal) that Gabor produced for 3 sec.

### Expectancy ratings

In all experiments, participants completed two contingency Likert scales to assess participants' expectations of the aversive image for each stimulus to ensure they learned the basic contingencies during training. They were shown the 90° and the 0° Gabor patches and asked to rate how likely the aversive image (or loud noise in Experiment 4) is to follow them. The scales ranged from 1 to 9 with 1 being “will not follow” and 9 being “most certainly will follow.” They also answered a question about how aversive the aversive image (or loud noise in Experiment 4) was, with 1 being not aversive and 9 being extremely aversive. Additionally, in Experiment 5, the participants were also asked to rate the novel test Gabor images as well as the CS+ and the CS−. They were then thanked for their time and the experiment ended.

### Data analyses

In the training phase, the effect of the stimulus was examined by comparing responses to the stimulus set that was paired with the aversive outcome (CS+) with responses to the one that was not (CS−), as a function of training trials. This was achieved by conducting a 2 (Stimulus; CS+ vs. CS−) × 15 (Trials; 1–15) repeated-measures ANOVA. Furthermore, during training, we also analyzed the number of safety signals participants produced over the 15 CS+ trials. Because safety signal presentation is a binary outcome (0 or 1) and this violates the assumptions of parametric statistics, we analyzed five blocks of three trials with a one-way ANOVA assessing the effect of Blocks (1–5).

Responses in the test phase assessed the effect of the stimulus to determine whether participants responded similarly to the stimulus that produced the trained safety signal versus the control stimulus, over eight test trials. This was achieved by conducting a 2 (Signal; Trained Safety Signal vs. Control [No Signal or New Signal]) × 8 (Trials 1–8) repeated-measures ANOVA. Furthermore, in the test phase for Experiments 2, 3, and 5, we compared the overall number of trained safety signals participants produced during the test compared to the new safety signal. This was achieved with a paired *t*-test using the total number of trained safety signals produced versus the new safety signals produced. Lastly, the expectancy of the CS+ and CS− (and the two test stimuli in Experiment 5) was assessed using paired *t*-tests.

## Supplementary Material

Supplement 1

## References

[LM053914FISC1] Angelakis I, Austin JL. 2015. Maintenance of safety behaviors via response-produced stimuli. Behav Modif 39: 932–954. 10.1177/0145445515610314 26463997

[LM053914FISC2] Cassaday HJ, Muir C, Stevenson CW, Bonardi C, Hock R, Waite L. 2023. From safety to frustration: the neural substrates of inhibitory learning in aversive and appetitive conditioning procedures. Neurobiol Learn Mem 202: 107757. 10.1016/j.nlm.2023.107757 37044368

[LM053914FISC08] De Kleine RA, Hutschemaekers MHM, Hendriks GJ, Kampman M, Papalini S, Van Minnen A, Vervliet B. 2023. Impaired action-safety learning and excessive relief during avoidance in patients with anxiety disorders. J Anxiety Disord 96: 102698. 10.1016/j.janxdis.2023.102698 37004425

[LM053914FISC3] Denny MR. 1971. Relaxation theory and experiments. In Aversive conditioning and learning (ed. Brush FR), pp. 235–295. Academic Press, New York.

[LM053914FISC09] Dickinson A, Dearing MF. 1979. Appetitive–aversive interactions and inhibitory processes. In Mechanisms of learning and motivation: a memorial volume to Jerzy Konorski (ed. Dickinson A, Boakes RA), pp. 203–231. Hillsdale Erlbaum, NJ.

[LM053914FISC4] Dinsmoor JA. 1977. Escape, avoidance, punishment: Where do we stand? J Exp Anal Behav 28: 83–95. 10.1901/jeab.1977.28-83 16812016 PMC1333616

[LM053914FISC5] Dinsmoor JA, Sears GW. 1973. Control of avoidance by a response-produced stimulus. Learn Motiv 4: 284–293. 10.1016/0023-9690(73)90018-0

[LM053914FISC6] Fernando AB, Urcelay GP, Mar AC, Dickinson A, Robbins TW. 2014a. Safety signals as instrumental reinforcers during free-operant avoidance. Learn Mem 21: 488–497. 10.1101/lm.034603.114 25135197 PMC4138357

[LM053914FISC7] Fernando AB, Urcelay GP, Mar AC, Dickinson TA, Robbins TW. 2014b. The role of the nucleus accumbens shell in the mediation of the reinforcing properties of a safety signal in free-operant avoidance: dopamine-dependent inhibitory effects of d-amphetamine. Neuropsychopharmacology 39: 1420–1430. 10.1038/npp.2013.337 24336447 PMC3988545

[LM053914FISC04] Flores A, López FJ, Vervliet B, Cobos PL. 2018. Intolerance of uncertainty as a vulnerability factor for excessive and inflexible avoidance behavior. Behav Res Ther 104: 34–43. 10.1016/j.brat.2018.02.008 29524740

[LM053914FISC8] Gillan CM, Urcelay GP, Robbins TW. 2016. Associative theories of avoidance behaviour. In The Wiley Blackwell handbook on the cognitive neuroscience of learning (ed. Honey R, Murphy RA), pp. 442–467. Wiley, Hoboken, NJ.

[LM053914FISC9] Graham BM, Milad MR. 2011. The study of fear extinction: implications for anxiety disorders. Am J Psychiatry 168: 1255–1265. 10.1176/appi.ajp.2011.11040557 21865528 PMC4118766

[LM053914FISC01] Konorski J. 1967. Integrative activity of the brain: an interdisciplinary approach. University of Chicago Press.

[LM053914FISC02] Leknes S, Lee M, Berna C, Andersson J, Tracey I. 2011. Relief as a reward: hedonic and neural responses to safety from pain. PloS One 6: e17870. 10.1371/journal.pone.0017870 21490964 PMC3072382

[LM053914FISC03] Leng L, Beckers T, Vervliet B. 2023. What a relief! The pleasure of threat avoidance. Emotion. 10.31234/osf.io/tnhu4 37971851

[LM053914FISC10] Lohr JM, Olatunji BO, Sawchuk CN. 2007. A functional analysis of danger and safety signals in anxiety disorders. Clin Psychol Rev 27: 114–126. 10.1016/j.cpr.2006.07.005 16997437

[LM053914FISC05] Lovaas I, Newsom C, Hickman C. 1987. Self‐stimulatory behavior and perceptual reinforcement. J Appl Behav Analysis 20: 45–68. 10.1901/jaba.1987.20-45 PMC1285951 3583964

[LM053914FISC12] Mackintosh NJ. 1974. The psychology of animal learning. Academic Press, New York.

[LM053914FISC13] Mineka S. 1979. The role of fear in theories of avoidance learning, flooding, and extinction. Psychol Bull 86: 985. 10.1037/0033-2909.86.5.985

[LM053914FISC14] Mowrer OH. 1951. Two-factor learning theory: summary and comment. Psychol Rev 58: 350. 10.1037/h0058956 14883248

[LM053914FISC15] Perez OD, Dickinson A. 2024. Dual-system free-operant avoidance: extension of a theory. *J Exp Psychol Anim Learn Cogn*. 10.1037/xan0000377 38661631

[LM053914FISC16] Rescorla RA. 1969. Establishment of a positive reinforcer through contrast with shock. J Comp Physiol Psychol 67: 260–263. 10.1037/h0026789 5785614

[LM053914FISC06] San Martín C, Jacobs B, Vervliet B. 2020. Further characterization of relief dynamics in the conditioning and generalization of avoidance: effects of distress tolerance and intolerance of uncertainty. Behav Res Ther 124: 103526. 10.1016/j.brat.2019.103526 31778930

[LM053914FISC17] Sangha S, Diehl MM, Bergstrom HC, Drew MR. 2020. Know safety, no fear. Neurosci Biobehav Rev 108: 218–230. 10.1016/j.neubiorev.2019.11.006 31738952 PMC6981293

[LM053914FISC18] Scholten WD, Batelaan NM, van Balkom AJ, Penninx BW, Smit JH, van Oppen P. 2013. Recurrence of anxiety disorders and its predictors. J Affect Disord 147: 180–185. 10.1016/j.jad.2012.10.031 23218248

[LM053914FISC07] Seymour B, O'Doherty JP, Koltzenburg M, Wiech K, Frackowiak R, Friston K, Dolan R. 2005. Opponent appetitive-aversive neural processes underlie predictive learning of pain relief. Nature Neurosci 8: 1234–1240. 10.1038/nn1527 16116445

[LM053914FISC19] Spielberger CD, Craighead WE. 2010. State-trait anxiety inventory. In The Corsini encyclopedia of psychology, Vol. 4, pp. 1698–1699. Wiley, Hoboken, NJ.

[LM053914FISC20] Urcelay GP. 2012. Exposure techniques: the role of extinction learning. In Exposure therapy: rethinking the model—refining the method (ed. Neudeck P, Wittchen H-U), pp. 35–63. Springer, New York, NY.

[LM053914FISC21] Urcelay GP, Prével A. 2019. Extinction of instrumental avoidance. Curr Opin Behav Sci 26: 165–171. 10.1016/j.cobeha.2019.01.018

[LM053914FISC22] Urcelay GP, Symmons K, Amos B, Toutounji H, Prével A. 2024. Renewal of instrumental avoidance in humans. J Exp Psychol Anim Learn Cogn 50: 197–209. 10.1037/xan0000383 39101917

[LM053914FISC23] Vervliet B, Indekeu E. 2015. Low-cost avoidance behaviors are resistant to fear extinction in humans. Front Behav Neurosci 9: 351. 10.3389/fnbeh.2015.00351 26733837 PMC4689807

[LM053914FISC24] Vervliet B, Lange I, Milad MR. 2017. Temporal dynamics of relief in avoidance conditioning and fear extinction: experimental validation and clinical relevance. Behav Res Ther 96: 66–78. 10.1016/j.brat.2017.04.011 28457484

[LM053914FISC25] Weisman RG, Litner JS. 1969. Positive conditioned reinforcement of Sidman avoidance behavior in rats. J Comp Physiol Psychol 68: 597. 10.1037/h0027682

