# Supplementary material for: Safety signals reinforce instrumental avoidance in humans
Source: Learn Mem. 2024 Aug;31(8):a053914. doi: 10.1101/lm.053914.123 (PMC11407691; doi:10.1101/lm.053914.123)
Supplement: Supplement 1 [file Supplementary_Material.docx]

**Safety signals reinforce instrumental avoidance in humans**

**Courteney T. L. Fisher & Gonzalo P. Urcelay.**

**Supplementary materials**


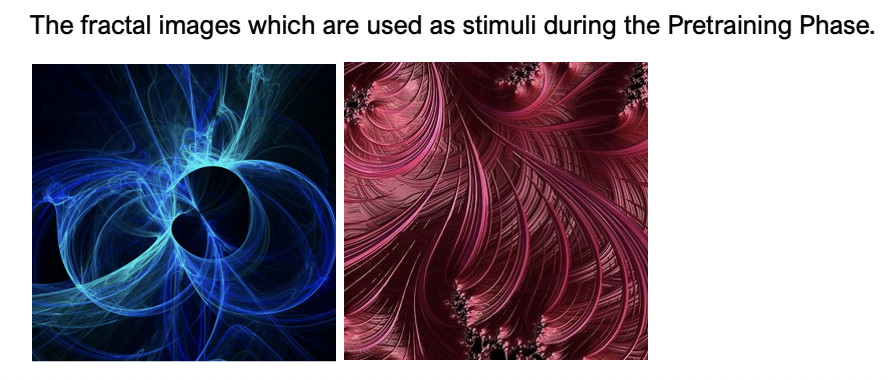


**Figure S1**

*The fractal images used for pre training.*

One was always followed by the aversive stimulus (aversive image Experiments 1-3 and 5; aversive sound Experiment 4).

**Instructions for the experiments: Experiments 1-4 had the same instructions except in experiment 4 they said, ‘loud noise’ in place of ‘aversive image’.**

*Pre Training:*

In the first phase of the experiment, you will see different abstract images that will be presented on the screen. Some of these will be followed by an unpleasant image and some will not. In other words, some of these abstract images predict the unpleasant images whereas other do not.

You can prevent the unpleasant images from appearing on the screen by pressing the space bar on your keyboard.

You may need to press the space bar multiple times to prevent the unpleasant images from appearing on the screen. You can press the spacebar as many times as you would like, but only some presses will prevent the unpleasant images from appearing on the screen.

Remember, your task is to try to prevent the unpleasant images from appearing on the screen.
Please click anywhere to continue

*Training instructions:*

The next phase is similar as the previous phase, except that the abstract images are now different. Some of these will be followed by an unpleasant image and some will not. Your task remains the same.

You can avoid the unpleasant images from appearing on the screen by pressing the space bar on your keyboard.
You may need to press the space bar multiple times to prevent the unpleasant images from appearing on the screen. You can press the spacebar as many times as you would like, but only some presses will prevent the unpleasant images from appearing on the screen.

Remember, your task is to try to prevent the unpleasant images from appearing on the screen.
Please click anywhere to continue

**Experiment 5 had additional instructions for the test phase of the experiment due to the response changing.**

*Test Instructions:*

The response will now no longer be the spacebar. Instead you will have to click the abstract image on the screen with your mouse. You may need to click more than once. Your task remains the same.

Click to continue

**Aversive Images**

For experiments 1-3 and 5 we used IAPS images as the aversive outcome. Participants were shown all six images on the screen and had to rank them from least to the most aversive. Table S1 includes a description of the image, the IAPS number, arousal and valance ratings for each image.

**Table S1**

| Description | IAPS | Valance Mean | Valance SD | Arousal Mean | Arousal SD |
| --- | --- | --- | --- | --- | --- |
| Vomit | 9325 | 1.89 | 1.23 | 6.01 | 2.54 |
| Spider | 1202 | 3.35 | 1.77 | 5.94 | 2.17 |
| Snake | 1050 | 3.46 | 2.15 | 6.87 | 1.68 |
| Toilet | 9301 | 2.26 | 1.56 | 5.28 | 2.46 |
| Cockroach | 7380 | 2.46 | 1.42 | 5.88 | 2.44 |
| Surgery | 3213 | 2.96 | 1.94 | 6.57 | 1.99 |

In all the experiments we used two fractal images to train the participants on how to use the task. We incorporated this aspect to the task so that we could try and remove any participants that don’t engage in the task early on. Figure 1 shows the fractal images we used.

**Anxiety analyses**

Anxiety scores were collected from all participants in each experiment. In a previous meta-analysis, it highlighted that trait anxiety is the most used in the literature for assessing anxiety and fear conditioning. We therefore used these scores and divided our participants into high and low anxiety scorers via a median split. We used this as a between subject’s variable and completed the same analysis as stated in the article.

**Experiment 1**

**Training avoidance behaviour**

There was no difference in avoidance responses for the CS+ and the CS- (see Figure S2A) *F*(1, 70) = .019, *p* = .89. Furthermore those with high and low anxiety made a similar amount of avoidance responses, *F*(1,70) = 2.23, *p* = .140. There was a main effect of trials *F*(3.664, 256.481) = 10.01, *p* < .001, ηp^2^ = .125. There was no interaction between anxiety and trials *F*(3.664, 256.48) = .603, *p* = .646; or between CS and anxiety *F*(1, 70) = .044, *p* = .834. Finally, there was no triple interaction between anxiety, CS and trials *F*(8.091, 566.341) = 1.103, *p* =.139.

**Number of safety signals produced**

As the trials increased participants produced more safety signals, *F*(10.59, 741.59) = 7.256, *p* <.001, ηp^2^ = .094. However, there was no difference between those with high and low anxiety scores, *F*(1,70) = .002, *p* = .926. Furthermore, there was no interaction between anxiety and number of safety signals produced *F*(10.594, 741.59) = 1.043, *p* = .304.

**Test data**

Those with high anxiety scores made more avoidance response than those with low anxiety scores, *F*(1,70) = 3.963, *p* = .05, ηp^2^ = .054 (see Figure S2B). Also participants with made more responses to the Gabor that produced the safety signal compared tot eh Gabor that produced nothing, *F*(1,70) = 6.312, *p* = .014, ηp^2^ = .083. Also as the trials increased the participants made fewer avoidance responses, *F*(3.499, 244.922) = 4.916, *p* <.001, ηp^2^ = .066. Furthermore, there was and no interactions between signal and trials *F*(4.788, 335.141) = 1.431, *p* = .215, ηp^2^ = .020; or between trial and anxiety *F*(3.499, 244.922) = 1.431, *p* = .215 or signal and anxiety *F*(1,70) = .846, *p* = .356. Finally, there was also no triple interaction *F*(4.788, 335.141) = 1.45, *p* = .208.

**Experiment 2**

**Training avoidance behavior**

In Experiment 2 a mixed ANOVA revealed that there was no difference in avoidance responses during training for high and low anxiety scorers, *F*(1,53) = 1.303, *p* = .259, ηp^2 =^.795 (see Figure S3a). Participants made more responses to the CS+ compared to the CS-, *F*(1, 53) = 7.312, *p* = .009, ηp^2^ = .121. There was also a main effect of trials *F*(7.05, 373.591) = 5.602, *p* <.001. Furthermore, participants made more responses to the CS+ compared to the Cs_ as trials progressed which was shown through an interaction, *F*(8.66, 459.305) = 3.225, *p* <.001, ηp^2^ = .057. However there was no interaction between anxiety and trials *F*(7.05) = .713, *p* = .762, ηp^2^ = .013, or between CS and anxiety scores *F*(1,53) = .762, *p* = .387, ηp^2^ = .014. Finally, there was also no triple interaction between anxiety scores, CS and trials *F*(8.66, 459.305) = .515, *p* = .858, ηp^2^ =.01.

**Number of signals produced**

As the trials increased participants produced more safety signals, *F*(10.344, 548.221) = 8.646, *p* <.001, ηp^2^ = .140. However, there was no difference between high and low anxiety scorers in the number of safety signals they produced, *F*(1,53) = .001, *p* = .972. Also there was no interaction between trials and anxiety *F*(10.344, 548.221) = .575, *p* = .840, ηp^2^ = .011.

**Test Data**

There was no difference in avoidance behavior between the Gabor that produced the trained safety signal and the Gabor that produced a new signal, *F*(1,53) = .093, *p* = .757, ηp^2^ = .002 (see Figure S3B). Critically, those with high anxiety and low anxiety made the same amount of responses during the test *F*(1,53) =.000, *p* = .985, ηp^2^ = .000. Participants did make less responses as the trials increased suggesting extinction *F*(3.793, 178.049) = 3.793, *p* = .009, ηp^2^ = .067. Furthermore there was no interaction between safety signal and anxiety *F*(1,53) = .352, *p* = .556, ηp^2^ = .007. or anxiety and trials *F*(3.359, 178.049) = 1.487, *p* = .216, ηp^2^ = .027. Furthermore, there was no interaction between Signal and trials *F*(5.194, 275.296) = 1.447, *p* = .205, ηp^2^ =.027 and there was no interaction between anxiety, signal and trials *F*(5.194, 275.296) = 1.935, *p* = .086, ηp^2^ = .035.

**Experiment 3**

**Training data**

Those with high anxiety made more responses compared to those with low anxiety scores, *F*(1,72) = 5.461, *p* = .022, ηp^2^ =.07 (see Figure S4A). Participants made more responses to the CS+ compared to the CS- as trials increased as shown by an interaction between trials and signal *F*(7.885, 567.742) = 5.360, *p* <.001, ηp^2^ = .069. Furthermore this was highlighted with main effects of CS, *F*(1,72) = 31.342, *p* <.001, ηp^2^ = .303 and trials *F*(7.975, 574.223) = 9.153, *p* <.001, ηp^2^ = .113. However, there was no interaction between signal and anxiety *F*(1,72) = .104, *p* = .748, .001. or between trials and anxiety *F*(7.975,574.223) = 2.067, *p* = .037, ηp^2^ = .028. There was also no interaction between signal, anxiety and trial *F*(7.885, 567.742) = 1.492, *p* = .158, ηp^2^ = .02.

**Number of signals produced**

Participants produced more safety signals as the trials increased, *F*(9.166,659.950) = 12.637, *p* <.001, ηp^2^ = .149. However, there was no difference in number of safety signals produced for high and low anxiety scorers, *F*(1,72) = .587, *p* = .446, ηp^2^ = .008. Moreover there was no interaction between anxiety and trials *F*(9.166,659.950) = .892, *p* = .533, ηp^2^ = .012.

**Test Data**

Critically, there was no difference in avoidance responses for the Gabor that produced the trained safety signal compared to the Gabor that produced the new novel safety signal, *F*(1,72) = 1.385, *p* = .243, ηp^2^ .019 (see Figure S4B). Furthermore, there was no difference between high and low anxiety scorers, *F*(1,72) = .815, *p* = .37, ηp^2^ = .011. As trials increased participants made fewer avoidance responses as shown by a main effects of trials, *F*(3.901, 280.899) = 4.763, *p* <.001. There was no interaction between anxiety and signal *F*(1,72) = .053, *p* = .819, ηp^2^ = .019; or between signal and trials *F*(5.058, 364.202) = .836, *p* = .558, ηp^2^ = .011. Moreover, there was no interaction between anxiety and trials *F(*3.901, 280.899) = .800, *p* = .531, .011; and there was no interaction between anxiety, signal and trials *F*(5.058,364.202) = 2.02, *p* = .074, ηp^2^ = .027.

**Experiment 4**

**Training Avoidance Behaviour**

There were no differences in avoidance behavior for the CS+ compared to the CS- *F*(1,39) = 2.788, *p* = .103 (see Figure S5A). Furthermore, there was no difference in avoidance behavior for high and low anxiety scorers, *F*(1,39) = 2.31, *p* = .137. As the trials increased the participants made more responses to the CS+ compared to the CS- as revealed by interaction, *F*(4.866, 189.787) = 5.347, *p* <.001, ηp^2^ = .121. Moreover, there was an effect of trials *F*(3.349, 130.61) = 8.232, *p* <.001, ηp^2^ = .174.

Furthermore, there was no interaction between CS and anxiety scores, *F*(1,39) = .015, *p* = .903; or between trials and anxiety *F*(3.349, 130.61) = 1.124, *p* = .344. Finally, there was no interaction between anxiety, CS and trials, *F*(4.866, 189.787) = 1.23, *p* = .280.

**Number of safety signals produced**

As the trials increased the participants produced more safety signals, *F*(5.624, 219.341). Those with high and low anxiety produced the same amount of safety signals *F*(1,39) = .609, *p* = .44. Furthermore there was no interaction between anxiety and number of safety signals produced *F*(5.624,219.34) = 1.9, *p* = .087.

**Test Data**

Those with low anxiety made more avoidance responses than the high anxiety people *F*(1,39) = 5.076, *p* = .03, ηp^2^ = .115 (see figure S5B). Participants also made less avoidance responses as the trials increased*, F*(2.763, 107.767) = 6.599, *p* < .001, ηp^2^ = .145. Participants made the same amount of avoidance responses to the Gabor that produced the safety signal and the Gabor that produced nothing, *F*(1,39) = 1.962, *p* = .169. Furthermore, there was no interaction between trials and signal, *F*(4.447, 173.44) = .722, *p* = .592; or between trials and anxiety, *F*(2.763, 107.767) = 6.599, *p* = .063. Moreover there was no interaction between signal and anxiety, *F*(1,39) = .949, p = .336; finally, there was no triple interaction, *F*(4.447, 173.44) = .447, *p* = .794.

**Experiment 5**

**Training Avoidance Behaviour**

Those with high anxiety made the same responses as those with low anxiety scores, *F*(1,54) = .038, *p* = .846 (see Figure S6A). Participants made more responses to the CS+ compared to the CS- as trials increased as shown by an interaction between trials and signal *F*(8.781, 456.624) = 5.384, *p* <.001, ηp^2^ = .094. Furthermore, this was highlighted with main effects of CS, *F*(1,54) = 39.01, *p* <.001, ηp^2^ = .429 and trials *F*(7.452, 387.51) = 2.536, *p* = .013, ηp^2^ = .047. There was an interaction between signal and anxiety *F*(1,52) = 4.51, *p* = .038, .08. But there was no interaction between trials and anxiety *F*(7.452, 387.513) = 1.027, *p* = .423. There was also no interaction between signal, anxiety and trial *F*(8.781, 456.63) = 1.75, *p* = .078.

**Number of safety signals produced**

As the trials increased the participants produced more safety signals, *F*(9.14, 475.48) = 3.94, p <.001. Those with high and low anxiety produced the same amount of safety signals *F*(1,52) = 2.74, *p* = .104. Furthermore, there was no interaction between anxiety and number of safety signals produced *F*(9.14, 475.48) = .905, *p* = .522.

**Test Data**

Critically, there was no difference in avoidance responses for the Gabor that produced the trained safety signal compared to the Gabor that produced the new novel safety signal, *F*(1,52) = 3.01 *p* = .089 (see Figure S6B). Furthermore, there was no difference between high and low anxiety scorers, *F*(1,52) = 2.877, *p* = .096.

As trials increased participants made the same number of avoidance responses as there was no main effect of trials, *F*(3.537, 183.93) = .682, *p* = .587. There was no interaction between anxiety and signal *F*(1,52) = 1.01, *p* = .320, ηp^2^ = .019; or between signal and trials *F*(3.8623, 200.845) = .554, *p* = .690. Moreover, there was no interaction between anxiety and trials *F(3.54, 183.931*) = 1.409, *p* = .237; and there was no interaction between anxiety, signal and trials *F*(3.682,200.845) = 1.873, *p* = .119.

**Combined data**

**Training Data for the online Experiments 1, 2, 3 and 5**

When assessing all of the training data together for participants in Experiments 1, 2, 3 and 5, they make more responses to the CS+ than the CS- as the trials increase, *F*(10.944, 2781.59) = 12.716, *p* < .001, ηp^2^ = .048 (*see Figure S7*). Furthermore, those with high anxiety made the same amount of avoidance responses as those with low anxiety, *F*(1,253) = 3.405, *p* = .066 although there was a tendency for higher responding in participants with high anxiety. Furthermore, there were main effects of CS, *F*(1,253) = 85.605, *p* < .001, ηp^2^ = .253; and trials, *F*(8.837, 2235.88) = 14.122, *p* <.001, ηp^2^ = .053. Moreover, there was no interaction between anxiety and trials, *F*(8.837, 2235.88) = .959, *p* = .471. However, there was no interaction between anxiety and CS, *F*(1,253) = .496, *p* = .482; and there was no triple interaction between CS, trials, and anxiety *F*(10.944, 2781.59) = .743, *p* = .697.

**Figures**

**Figure S2**

Results from Experiment 1 for high and low anxiety scorers *(A)* Training data depicting avoidance responses to the CS+ and CS-. Each block contains 3 trials.  *(B)* Test data comparing the trained signal to no signal.

**Figure S3**

Results from Experiment 2 for high and low anxiety scorers  *(A)* Training data depicting avoidance responses to the CS+ and CS-. Each block contains 3 trials. *(B)* Test data comparing the trained signal to a new signal.

 

**Figure S4**

Results from Experiment 3 for high and low anxiety scorers *(A)* Training data depicting avoidance responses to the CS+ and CS-. Each block contains 3 trials. *(B)* Test data comparing the trained signal to a new signal.

**Figure S5**

Results from Experiment 4 for high and low anxiety scorers *(A)* Training data depicting avoidance responses to the CS+ and CS-. Each block contains 3 trials. *(B)* Test data comparing the trained signal to no signal.

**Figure S6**

Results from Experiment 5 for high and low anxiety scorers *(A)* Training data depicting avoidance responses to the CS+ and CS-. Each block contains 3 trials. *(B)* Test data comparing the trained signal to a new signal.

**Figure S7**

Results from the training data for Experiment 1-3 and 5 for high and low anxiety scorers  Training data depicting avoidance responses to the CS+ and CS-. Each block contains 3 trials. Participants respond more to the CS+ compared to the CS- and those with high anxiety make more avoidance responses to both the CSs compared to the low anxiety scorers.
